# Chitinase is stored and secreted from the inner body of microfilariae and has a role in exsheathment in the parasitic nematode *Brugia malayi*

**DOI:** 10.1016/j.molbiopara.2008.06.007

**Published:** 2008-09

**Authors:** Yang Wu, Gillian Preston, Albert E. Bianco

**Affiliations:** Molecular and Biochemical Parasitology, Liverpool School of Tropical Medicine, Pembroke Place, Liverpool L3 5QA, UK

**Keywords:** MF (microfilariae), L3 (infective third-stage larva), kDa (kilodalton), SDS-PAGE (SDS-polyacrylamide gel electrophoresis)

## Abstract

Chitinase expression in microfilariae of the parasitic nematode *Brugia malayi* (*B. malayi*, *Bm*) is coincidental with the onset of their infectivity to mosquitoes. An antibody raised to *Onchocerca volvulus* (*O. volvulus*, *Ov*) infective-stage larval chitinase (*Ov*-CHI-1) was specifically reactive against *B. malayi* microfilarial chitinase and was used to study the localization of chitinase in *B. malayi* during microfilarial development and transmission to the insect vector. Immuno-electron microscopy (IEM) was used to demonstrate that the chitinase was confined to the inner body of the microfilariae and furthermore that chitinase was only present in sheathed microfilarial species, although the inner body is present in all species. Observation using the IEM implicates two distinct routes of chitinase secretion from the inner body, via either the pharyngeal thread, or during transmission of the microfilariae to the vector, contained in vesicle-like structures. Many morphological studies have described the structure of the inner body, but no function has been assigned to it as of yet. Although it has been commented that the cells surrounding the inner body and pharyngeal thread are those destined to become the intestine and pharynx and that the inner body represents a store of material. Our studies suggest that chitinase is one such product stored in the inner body and that it is secreted during the exsheathment of the microfilaria in the mosquito.

## 1 Introduction

Lymphatic filariasis is a complex of diseases caused by infection with the parasitic nematodes, *Brugia malayi*, *Wuchereria bancrofti* and *Brugia timori*. It is still a significant source of chronic morbidity throughout the tropics causing conditions such as elephantiasis and tropical pulmonary eosinophilia, with more than 130 million people currently estimated to be infected. In these mosquito-borne diseases, microfilariae (MF) and infective third-stage larvae (L3) are of fundamental importance to the transmission and continuation of parasite life cycles. Deeper insights into the molecules and factors involved in governing the development and infectivity of these stages could potentially lead to the discovery of new targets for transmission-blocking therapy.

Chitinases are enzymes that catalyze the hydrolysis of beta-1, 4-*N*-acetyl-d-glucosamine linkages in chitin polymers. There are various chitinase enzymes present in many living organisms from bacteria to man, with roles in cell wall modification, carbon source degradation and defense against pathogens [1–4]. In protozoa, chitinases have been shown to facilitate the transmission between the vector and the host [5,6]. In filarial parasites, chitinases have been identified and characterized as stage-specific molecules in several species [7–9]. For example two stage-specific chitinase genes from *Onchocerca volvulus* (accession number L42-21) and *B. malayi* (accession number M73689), share 81% similarity at the amino acid level. These have both been designated as glycosyl hydrolases family 18 (GH18), however they differ in their expression. *Bm*-chitinase is expressed in the MF stage, *Ov*-chitinase in the infective L3 stage.

Vaccination studies have highlighted the importance of chitinases in host protection [8], the protective potential of filarial chitinase originally recognized by Fuhrman et al. [8,10]. Fuhrman found that the epitope of *B. malayi* chitinase was recognized by a monoclonal antibody, MF1, as well as by the sera of putative immune immigrants who had remained amicrofilaremic for 3–6 years after arriving in an area where Brugian filariasis was endemic [11,12]. Furthermore, it was reported that in bancroftian filariasis, the reactivity of the MF1 epitope was found to be inversely correlated with observed levels of microfilariae [13]. *In vivo*, passive transfer of the monoclonal antibody MF1 was shown to result in transiently reduced microfilaremia in *B. malayi* infected jirds [10]. MF1 antigen has also been reported to promote cell adherence to the microfilarial surface and its subsequent killing [8,10]. The protective MF1 epitope of chitinase was discovered to be located close to its carboxyl terminus, consisting of the last 52 amino acids of the protein [14]. Interestingly the microfilarial protein recognized by MF1 was only found to be present after the microfilariae had matured for several days in the vertebrate host with it being barely detectable in microfilariae collected within 2 days of birth [12]. Thus the appearance of chitinase in the microfilariae is coincident with the onset of their infectivity to the mosquito.

Because of chitinases’ chitinolytic activities, many studies have attempted to address the function of chitinases on chitin containing structures, notably, the eggshell or eggshell-derived structures such as filarial sheath. In an egg hatch assay of *Heligmosomoides polygyrus*, chitinase activity was found to increase with the age of the eggs and was released into the medium as the eggs hatched [15]. These activities were shown to be very sensitive to inhibition by the chitinase inhibitor allosamidin, with an IC50 for crude egg extract of 2.2 nM. In this assay allosamidin slowed, but did not abolish hatching of the eggs. Chitinases of *Onchocerca gibsoni* adult worms were also purported to degrade the chitinous oolema surrounding the developing eggs *in utero* [16]. There has been no direct evidence to ascertain the function of nematode stage-specific chitinases, however some theories have been put forward. It has been proposed that *Brugia* spp. chitinase may be required for exsheathment within the arthropod vector [17] and that OV-CHI-1 may be a mediator of the ecdysis of the old chitin-based cuticle during moulting [18].

Midgut penetration of the MF in the mosquito is necessary for subsequent larval development within the insect. The interruption of transmission by interfering with the parasite molecules involved in penetration would be important in the development of transmission-blocking therapy. Sera from vaccinated animals which, were partially protected against challenge infection, reacted with chitinase from *O. volvulus* L3 larvae [7]. Vaccination of jirds with radiation-attenuated *Acanthocheilonema viteae* (*A. viteae*) larvae has been proven to be a potent way to induce partial protection, giving 67% protection against a challenge infection of *A. viteae* L3 larvae [19]. In an alternative model, DNA immunization with *O. volvulus* L3 larval chitinase induced statistically significant levels of protection against L3 challenge infection in mice [20].

The inner body of microfilariae is an amorphous structure in the mid region of the MF, situated between the excretory cell and G1 cell. Morphology and size of the inner body varies between species, from a large elongated continuous sac to a series of small isolated spheres ([21], Supplementary Fig. 1). Granules containing storage substances make up the bulk of the substance of the inner body. The inner body along with the pharyngeal thread is regarded to be potentially the precursor of the intestine. Currently there is very little information about the function of the inner body in the available literature. However, the transient nature of the inner body, being broken down within 2 days of transmission to the vector, make it worthy of further investigation. This transient inner body and the MF chitinase we believe is released upon its breakdown in *B. malayi* may play a role during the transition from life in the mammalian host to that in the vector. To confirm this hypothesis, a study was conducted to observe the expression patterns of MF chitinase during transmission. We attempted to identify the destination of any secreted MF chitinase, with the aim of elucidating the pathway of secretion during transmission of the MF to the arthropod vector and the biological function of this molecule in *B. malayi*.

## 2 Materials and methods

### 2.1 Parasites sources

Mongolian jirds (*Meriones unguiculatus*) were infected intra-peritoneally with adult *B. malayi* larvae obtained from TRS Labs (Athens, USA). Microfilariae were isolated by peritoneal lavage of infected animals and purified by sedimentation through a PD10 sepharose column. *B. malayi* L3 stage larvae were obtained from crushing mosquitoes (*Aedes aegypti*) 12–14 days after infection with microfilariae. *B. malayi* eggs were collected after dissection of adult females. Microfilariae of *A. viteae* were obtained from jird blood drawn by cardiac puncture under terminal anesthesia. *D. immitis* MF was extracted from infected cat blood (TRS Labs). *Loa loa* and *W. bancrofti*, MF were extracted from blood taken with consent from an infected individual in Kumba, Cameroon and from blood taken with consent from an infected patients in Jakarta, Indonesia, respectively. Microfilarae were filtered from the blood samples using a 3.0-μm pore size millipore membrane filter, washed in RPMI medium and concentrated by centrifugation (600 × *g* × 15 min). *O. volvulus* microfilariae examined were collected from skin snips obtained with consent from an infected individual in Kumba, Cameroon. To obtain post-transmission microfilariae, *A. aegypti* mosquitoes (Liverpool Blackeye strain) were infected by membrane feeding with *B. malayi* microfilariae suspended in rabbit blood at a concentration of 200,000 MF/ml. After 20 h, engorged females were collected, blood meals dissected and microfilariae recovered from the blood by applying the dissected, crushed blood meals to the surface of a 15 ml tube of lymphoprep, after centrifugation the microfilariae were collected as a pellet. Whole mosquitoes were also fixed 24 h post-infection to study the microfilariae *in vivo*.

### 2.2 Antibodies used in this research

Mono-specific antibodies raised against recombinant *Ov*-CHI-1 full-length protein were used in this research. Details of the recombinant proteins expression and production of antibodies have been described previously [18]. This antibody recognized recombinant proteins of both the N- and C-terminal domains of the intact *Ov*-CHI-1protein and is likely to contain a mixture of specificities cross-reacting amongst the family 18 glycosyl hydrolases [18].

### 2.3 Immmuno-electron microscopy (IEM)

The parasites of different species as previously mentioned were used for IEM according to methods described [22]. Anti-*Ov*-CHI-1 antiserum (diluted 1 in 750 in the block buffer) was used with goat-anti-rabbit colloidal gold conjugates of diameter 5, 10 or 20 nm (British Biocell) diluted 1 in 20 in block buffer to reveal antibody–antigen complexes. The grids were counterstained with a 2% aqueous uranyl acetate solution (5 min) and Reynolds lead citrate (1 min) and then examined using a Phillips CM10 transmission electron microscope.

### 2.4 Chitinase activity assay

One thousand microfilariae of each *A. viteae*, *B. malayi*, *D. immitis*, *L. loa*, *O. volvulus* and *W. bancrofti* were homogenised in 200 μl, ice-cold 0.1 M sodium acetate buffer (pH 6.2). The homogenate was centrifuged at 14000 × *g* for 10 min to remove insoluble matter. The supernatant was then assayed for chitinase activity using the method described by Robbins et al. [23]. Reactions containing soluble extract from 1000 microfilarae in 200 μl were set up with 20 μl of the substrate 4-methylumbelliferyl-*N*,*N*,*N*-trisaccharide (4-MU-(GlcNAc) 3) to give a final concentration of 20 μM. Reactions were incubated at room temperature for 4 h, then stopped by the addition of 1.5 ml of a 0.1-M glycine–NaOH solution (pH 10.4). The fluorescence was measured immediately at 350 nm excitation, 440 emission in a PerkinElmer Life Science Model L330 luminescence spectrometer. The standard curve was constructed using 4-methylbelliferone in glycine buffer. The chitinase activity was calculated as picomoles of 4-MU-(GlcNAc) 3 generated per 1000 microfilarae soluble protein, per hour at room temperature.

### 2.5 Exsheathment assay and allosamidin treatment

The exsheathment assay was adapted from that of Devaney and Howells [24] with some modifications. Briefly, purified *B. malayi* microfilariae were suspended in 50 μl of phosphate-free buffer in a cavity slide and incubated in the presence of varying concentrations of the specific chitinase inhibitor allosamidin (University of Tokyo) (0–500 nm) in multiples of five repeats. At the end of 2 h incubation, 1.5 μl of each of 2 M CaCl_2_ and 2 M MgSO_4_ were added to make a final concentration of 60 mM. The cavities were then sealed with coverslips and the slides incubated at 27 °C for a further 4 h before assessment of the level of exsheathment. Differential counts of sheathed and exsheathed microfilariae were carried out using fluorescent wheat germ agglutinin (WGA) assay as described [25]. Sheathed and exsheathed parasites were counted using an Olympus BX60 fluorescent microscope.

### 2.6 MTT assay of viability

To ascertain if the allosamidin was having a detrimental affect on larval viability, a MTT assay was carried out along with the exsheathment assay. *B. malayi* microfilariae (4000 per group) were incubated for 2 h with varying concentrations of allosamidin (0–500 nm), after which, MTT (3-[4,5-dimethylthiazol-2-yl]-2,5-diphenyltetrazoluim bromide) (Sigma) at a concentration of 5 mg/ml in PBS was added to give a final concentration of 0.5 mg/ml. The microfilariae was then incubated for a further one and a half hours at 37 °C, washed twice with PBS and then re-suspended in 200 μl of dimethyl sulphoxide (Sigma). The OD was measured at 490 nm immediately and after 1 h at room temperature.

### 2.7 Gel electrophoresis and immuno-blotting

Proteins were extracted from parasites by boiling for 5 min in electrophoresis sample buffer [18]. Insoluble material was removed by centrifugation for 5 min at 16000 × *g*. Extracts were fractionated on a 12.5% polyacrylamide gel using the Tris–glycine–SDS system. Separated proteins were then transferred via electrophoresis to nitrocellulose and immuno-blotted with anti-Ov-CHI-1 antisera at a dilution of 1/3500 using the method described previously [18]. Goat anti-rabbit IgG (H+L) horseradish peroxidase conjugate (Nordic 1/2000) was used to localize antibody–antigen complexes. The blot was developed using 0.05% (w/v) 3,3′ diaminobenzidine tetrahydrochloride solution (Sigma).

Excretory/secretory (ES) products were collected following chemically induced exsheathment of 50,000 *B. malayi* microfilariae suspended in 1 ml of phosphate-free buffer to which 60 mM CaCl_2_ and MgSO_4_ had been added. A control using same number of microfilariae in buffer without CaCl_2_ and MgSO_4_ was similarly set up. A plate exsheathment was also performed on the same batch MF as described above to assess their level of exsheathment. The ES products were concentrated using a protein concentrator column (cutoff 10,000 kDa MW, Sartorius) and the concentrated ES was mixed with SDS-PAGE electrophoresis sample buffer and boiled for 5 min. The proteins of ES products from 50,000 *B. malayi* microfilariae were then separated on a 12.5% denaturing gel along with a sample collected under the same conditions in phosphate-free buffer alone. The separated proteins were transferred electrophoretically to nitrocellulose and probed with anti-Ov-CHI-1 antibodies as described above.

## 3 Results

### 3.1 Reactivity of Ov-CHI-1 antisera against *B. malayi* chitinase and the localization of chitinase in the microfilariae

**Fig. 1:**
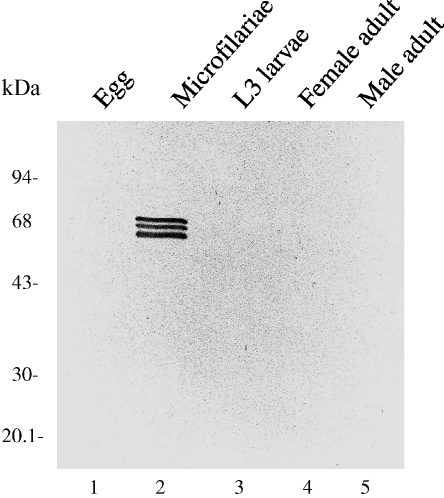
Stage-specific expression of filarial chitinases in *B. malayi*. (A) An immuno-blot of the *B. malayi* lifecycle, probed with antisera raised against *O. volvulus* L3 chitinase (Ov-CHI-1). Samples from left to right; lane 1, egg; lane 2, microfilariae; lane 3, L3; lane 4 adult female and lane 5, adult male. (B) An immuno-blot of the *B. malayi* lifecycle, probed with pre-immune sera from the rabbit in which the Ov-CHI-1 was raised. Samples as before from left to right; lane 1, egg; lane 2, microfilariae, lane 3, L3, lane 4, adult female; lane 5 adult male.

The antibody to Ov-CHI-1, raised against *O. volvulus* L3-chitinase in our laboratory was used to probe the proteins of *B. malayi* lifecycle by immuno-blot. It reacted with *Brugia* spp. without losing stage specificity and reacted only with the microfilarial stage in which the highly antigenic triplets of polypeptides (of ∼68–73 kDa) were detected (Fig. 1). An immuno-blot of the *B. malayi* lifecycle was also probed with the pre-immune sera from the same rabbit, and showed no reactivity (data not shown). These results were as previously reported [18] and consistent with the stage-specific characterization of the microfilarial chitinases described by Fuhrman et al. [8,12].

**Fig. 2:**
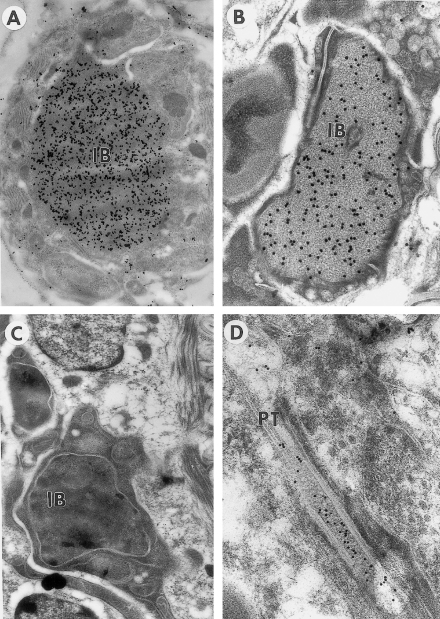
Localization of Ov-CHI-1 in microfilarial sections. Immunogold electron micrographs of: (A) the inner body of *B. malayi* microfilaria (23,000×) and (B) the inner body of *W. bancrofti* microfilaria (57,000×), both show chitinase labeling in this location. (C) Immunogold electron micrograph of the inner body of *D. immits* microfilaria (31,000×), a lack of chitinase labeling was seen in this species. (D) An immungold electron micrograph of the *B. malayi* pharyngeal thread (57,000×) showing chitinase within the lumen. Indicated are: IB, inner body; PT, pharyngeal thread.

Because of its specificity in its reaction to microfilarial chitinase of *B. malayi*, we used Ov-CHI-1 antibody to characterize the expression of Bm-MF chitinase using IEM. This showed that in *B. malayi*, chitinase was confined to the inner body of the microfilariae (Fig. 2A). Fig. 2A–C shows immuno-electron micrographs of the inner bodies of *B. malayi*, *W. bancrofti* and *D. immitis*, respectively. Chitinase labeling is clearly seen in the first two sheathed species but not in the latter, which is unsheathed. Chitinase labeling was also observed to be present within the lumen of the pharyngeal thread, the structure running from the inner body to the tip of the cephalic space (Fig. 2D).

**Fig. 3:**
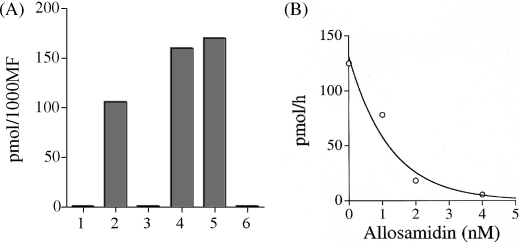
(A) Chitinase activity of microfilariae measured by fluorometry. Measured chitinase activity expressed in picomoles of substrate metabolized per 1000 MF of six microfilarial species, lane 1, *A. viteae*; lane 2, *W. bancroft*i; lane 3, *O. volvulus*; lane 4, *B. malayi*; lane 5, *L. loa*; and lane 6, *D. immitis*. (B) Chitinase activity of 1000 *B. malayi* MF measured by fluorometry using 4-methylumbelliferyl-trisaccharide as a substrate. The inhibition profile obtained with allosamidin, titrated in nM.

The microfilariae of *W. bancrofti*, *B. malayi* and *L. loa* are enclosed in a sheath derived from the original eggshell, whilst the microfilariae of *O. volvulus*, *A. viteae* and *Dirofilaria immits*, are unsheathed. IEM localization using Ov-CHI-1 antisera to probe sections of MF from these six filarial species (data not shown) demonstrated that only those MF having a sheath, (*B. malayi*, *W. bancrofti* and *L. loa*) stored chitinase within their inner bodies. Unsheathed species (*A. viteae*, *O. volvulus* and *D. immtis*) did not store chitinase, although they do also have an inner body. These results were confirmed by immuno-blot of the MF of these species (Fig. 3A).

### 3.2 Chitinase activity assays and inhibition by allosamidin

To confirm that chitinase was not present in the unsheathed species of microfilariae and to ascertain that the enzyme was active, we measured the chitinolytic activity of extracts obtained from 1000 MF of each of the six microfilarial species, using 4-MU-(GlcNAc) 3 as a substrate (Fig. 3A). Enzyme activity was found to be present only in the sheathed microfilariae of *L. loa*, *B. malayi* and *W. bancrofti* but not in the unsheathed microfilariae. This verifies that the sheathed microfilariae not only contained the chitinase but also possess chitinolytic activity. Chitinase activity of the MF could be inhibited in a dose dependant manner by the specific chitinase inhibitor allosamidin, with almost complete ablation at an allosamidin concentration of 4 nM (Fig. 3B).

### 3.3 Allosamidin inhibition in assay of *in vitro* exsheathment

**Fig. 4:**
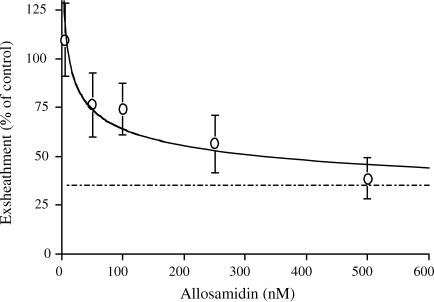
Exsheathment of *B. malayi* microfilariae, inhibition profile obtained with allosamidin. Chemically induced exsheathment of *B. malayi* MF in the presence of allosamidin, inhibition is expressed as a percentage of that seen in the control.

To determine whether allosamidin inhibits microfilariae exsheathment, an *in vitro* exsheathment assay was carried out in the presence of increasing concentrations of allosamidin (0–500 nM). Allosamidin reduced the exsheathment up to 65% (as compared to the observed level of control exsheathment) at the highest concentration of inhibitor at which microfilarial viability was not compromised (Fig. 4). Viability was ascertained by an MTT assay.

### 3.4 *B. malayi* infection of mosquitoes *in vivo*

**Fig. 5:**
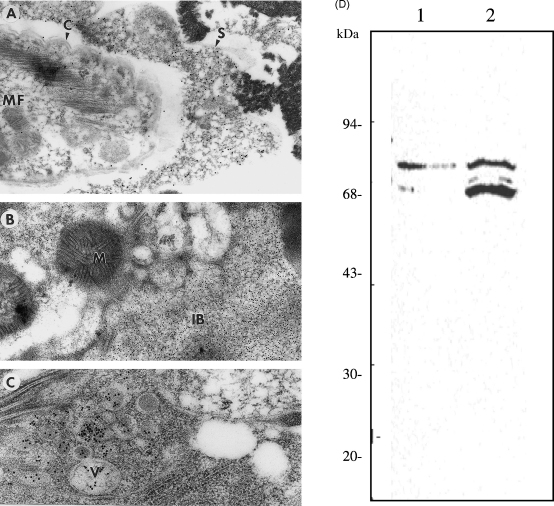
Localization chitinase in post-infective *B. malayi* microfilariae. (A) Immunogold electron micrograph of *B. malayi* microfilaria within bloodmeal inside *A. aegypti*, 20 h post-infection (28,750×), labeling of 10 nm colloidal gold can be seen between the microfilarial cuticle and sheath. (B) *B. malayi* microfilaria, 24 h post-infection, found between the peritrophic membrane and the abdomen wall of an infected mosquito. The chitinase labeling (5 nm colloidal gold) and structural changes of the inner body can be seen (52,500×). (C) Immuno-electron micrograph of *B. malayi* microfilaria fixed during chemically induced exsheathment *in vitro* (71,250×), chitinase labeled with 20 nm colloidal gold was observed to localize within vesicle-like structures. Indicated are: C, cuticle; IB, inner body; M, mitochondria; MF, microfilaria; S, sheath; V, vesicle. (D) Shows that chitinase presence in ES products of exsheathing *B. malayi* MF. Immuno-blot probed with antibody to Ov-CHI-1 on ES products of *B. malayi* microfilaria during chemically induced exsheathment *in vitro*. Samples from left to right are: lane 1, ES from a control population of *B. malayi* MF; lane 2 ES from *B. malayi* MF exsheathed *in vitro*.

Infection of mosquitoes with high numbers of *B. malayi* microfilariae using a membrane feeding system permitted the recovery of a usable number of 20 h, post-transmission MF, which were processed for IEM. These parasites, when probed with the Ov-chitinase antibody, showed that large amounts of chitinase accumulated in the space between the microfilariae cuticle and the sheath (Fig. 5A). No chitinase was observed in the pre-transmission microfilariae in this location. This intimates that shortly after transmission, chitinase was being secreted by the MF *in vivo*. Microfilariae, observed within mosquitoes 24-h post-infection, were found to be located between the peritrophic membrane and the abdominal wall. At this time the inner body was perceived to be less electron dense, having a sponge-like appearance and a poorly defined outline (Fig. 5B). IEM studies were also carried out on microfilariae after 4 h of chemically induced exsheathment. These showed the inner body to be disrupted and chitinase containing vesicles-like structures of varying size to be dispersed throughout the microfilarial body (Fig. 5C).

### 3.5 Immuno-blot of the ES of exsheathing *B. malayi* MF

To corroborate whether MF-chitinase was excreted during exsheathment, an immuno-blot with anti Ov-CHI-1 was performed on ES products collected from the supernatant of a concentrated population of 50,000 *B. malayi* microfilariae/ml undergoing *in vitro* exsheathment. A control population of same number of *B. malayi* microfilariae was incubated in buffer without CaCl_2_ and MgSO_4_. The results showed that although chitinase was present in the ES of both populations, the secretion of chitinase from *B. malayi* MF, which had been induced to exsheath was greater than that in the control MF (Fig. 5D).

## 4 Discussion

The antibody to Ov-CHI-1, raised against *O. volvulus* L3-chitinase specifically recognized the stage-specific chitinases of the MF of *Brugia* spp. Although both our and Fuhrmans’ groups have identified stage-specific chitinases in the MF of *B. malayi*, Raghavan et al. have also reported the presence of a 43-kDa chitinase in the L3 stage of *Brugia* [26]. Since the Ov-CHI-1 antibody was generated against *Onchocerca* third stage larvae, one might expect to observe some cross-reaction with the L3 stage. However, we have never observed this band in our immuno-blots of *B. malayi* L3 larvae. Furthermore no *B. malayi* L3 chitinolytic activity has ever been found in substrate gel assays carried out in our lab. Therefore we utilised the Ov-CHI-1 antibody in our investigations into *B. malayi* MF chitinases.

Localization of chitinase in *B. malayi* could be clearly observed by IEM, being present only in the inner body and pharyngeal thread of the MF. The pharyngeal thread appears to be in contact with the inner body and the chitinase detected within could be the result of a sustained discharge or leakage of chitinase. The inner body of microfilariae is an amorphous structure situated between the excretory cell and G1 cell, which exists as a single body or as several discrete masses. The inner body appears to have no limiting membrane; cells apposed to it have been observed to have their cytoplasm in contact with the substance. The morphology of the inner body may vary between species, from an elongated continuous sac, which may contain dense granules, to a series of many small isolated spheres. The inner body begins to develop soon after birth and is broken down within 48 h of transmission to the vector [27,28]. The fact that chitinase is present in the pharyngeal thread could implicate it as a route of transport for the discharge of inner body contents to the external surface of the larvae. The expression of MF chitinase starts practically as soon as the microfilariae were born and ceases within 2 days after transmission into the mosquito. We have discovered that the inner body is first observable by electron microscopy 2 days after birth, at this early stage in its development chitinase was already present. There has been very little information about the function of the inner body in available literature, but the timing of its creation and demise indicate that the products contained within it are likely to be of great importance in transmission of *B. malayi* to the vector and infectivity or adaptation to the host.

Chitinase expression in microfilariae was found to be restricted to those species having a microfilarial sheath. MF of *W. bancrofti*, *B. malayi* and *L. loa* have a sheath which is derived from the original eggshell, MF of *O. volvulus*, *A. viteae* and *D. immits*, are unsheathed. We found that although all six species have an inner body only in the sheathed species does it contain chitinase, as observed by IEM. These results were further corroborated by the immuno-blot on MF of these species. We have also confirmed that the chitinase present in sheathed species was active and could be inhibited by allosamidin. The presence of MF chitinase only in the inner bodies of sheathed species could be indicative that its function is one of exsheathment.

Exsheathment of Brugian microfilariae has been observed to occur mainly in the hemocoel of the mosquito but may occasionally occur within the midgut [29–31]. Exsheathment in the midgut may be the result of mechanical damage to the sheath by the pharyngeal armatures of the female mosquito [30]. Chitin is an important component of the sheath and it has been demonstrated that chitin synthesis is critical to sheath morphogenesis in *B. malayi* [11]. Diflubenzuron treatment of gravid females inhibited incorporation of radio labeled chitin precursors and resulted in progeny with morphologically aberrant sheaths. It is unknown why sheaths are retained in some species and not in others, but they could be of some biological importance in the vertebrate host, or be employed as a defense mechanism in the vector. Humoral encapsulation of both sheaths and microfilariae does occur in the hemocoel of the mosquito, involving the deposition of a pigmented material (melanin) on their surface within 30 min of reaching the hemocoel without any hemocyte participation [32]. Cast sheaths lying in the hemocoel could act to decoy the defense reaction in the insect host [30]. In experiments in which microfilariae were inoculated into the thorax of *A. aegypti*, the melanization response of the mosquitoes to chemically exsheathed microfilariae was significantly reduced when compared with that to the sheathed control [33].

*In vitro* exsheathment assays carried out in the presence of increasing concentrations of allosamidin (0–500 nM) showed an inhibition of exsheathment of 65% at the highest concentration of inhibitor at which microfilarial viability was not compromised. The inhibition of exsheathment appeared to be dose dependent between 0 and 600 nm, over this dose, the inhibition persisted without compromising viability. The percentage inhibition was expressed in relation to the level of exsheathment found in the control. The level of exsheathment in the control group was low at around 35%. Greater exsheathment could be obtained by using blood-derived MF (over 90%) instead of MF derived from the peritoneum, but these were unavailable to us in large enough numbers to complete the series of experiments. These results indicate that during *in vitro* exsheathment at least, BM-MF chitinase participates in the exsheathment of microfilariae. In an egg hatch assay of *H. polygyrus*, chitinase was found to be released into the medium when eggs hatched [15]. These activities were inhibited by allosamidin, which resulted in a delay in, but not a full inhibition of hatching. A re-count of exsheathment levels was carried out after 24 h further incubation at 27 °C, at which time no further exsheathment was observed indicating that allosamidin had indeed inhibited, rather than retarded exsheathment.

The early development of unsheathed microfilariae is similar to that of sheathed microfilariae, but the differences occur during later development. The convoluted eggshell observed to be present during the latter period of microfilarial development detaches from the uterine wall and stretches to form the sheath of the larva, in unsheathed species the mature microfilariae forces its way through the eggshells as opposed to molding it into a sheath [34]. Chitin has been demonstrated to be present in the eggshells of both *O. gibsoni* and *O. volvulus* [16]. The uterine chitinase of *O. gibsoni* has been found to play a role in hatching and in eggshell formation [35]. The chitin synthesis gene of *B. malayi* (*chs-1*) has been shown by *in situ* hybridization to be highly expressed in oocytes and zygotes, this expression diminishes with embryo age and is reported to be undetectable by the time of the C-stage embryos [36]. Chitin synthesis and eggshell formation commence after fertilization, the precursor *N*-acetyl glucosamine being obtained from glycogen stores. Chitinolytic activity may be essential in the release of the filarial larvae from the eggshell or sheath.

*In vivo*, exsheathment of *B. malayi* occurs after the microfilariae have been taken up by the feeding mosquito and the parasite has crossed the midgut into the hemocoel, this being location in which the majority of exsheathment occurs. *B. malayi* MF collected at 20 h post-transmission, when probed with the Ov-chitinase antibody, showed large amounts of chitinase had accumulated in the space between the microfilariae cuticle and the sheath, where no chitinase was observed pre-transmission. This suggests that chitinase was secreted soon after transmission by the microfilariae in *in vivo*. In MF observed by IEM within mosquitoes 24-h post-infection the inner body was found to be less electron dense, having a sponge-like appearance and a poorly defined outline. At this time chitinase is still present. If the chitinase and other inner body products are discharged solely via the pharyngeal thread remains unclear. In MF examined after 4 h of chemically induced exsheathment, the inner body was seen to be broken down into chitinase containing vesicles of varying size which were found to be dispersed through the microfilarial body. These vesicles could act as a mode of secretion post-transmission as an alternative to or in combination with that via the pharyngeal thread. It has been suggested that in the malaria parasite *Plasmodium gallinaceum* chitinase aids ookinete penetration of the chitin-containing peritrophic membrane of *A. aegypti* [5] and it is postulated that this could also be the case in *B. malayi* MF. However the fact that sheathed microfilariae has been observed in the hemocoel, where the majority of exsheathment is thought to occur indicates that this is unlikely.

During transmission of the MF to the mosquito it is likely that all the products contained within the inner body are released upon its demise and it is probable that other components stored within the inner body are likely to be also involved in the promotion of infectivity. MF-chitinase was found to be present in the ES products during exsheathment *in vitro*. Although some MF-chitinase was also present in the ES products of the control. This could be a result of the release of the chitinase that we have observed to be trapped between the microfilaria and the sheath post-transmission as the microfilariae break through, and escape from the sheath. Some chitinase signal would be expected even in the control owing to the presence of some dead or broken larvae in the large numbers used for this assay. The secreted forms in the ES were seen to be mainly of bands 1 and 3. It could be that these forms are more important in exsheathment or that the band 2 form is degraded during the exsheathment assay.

The breakdown of the inner body upon transmission to the vector parallels the situation seen in the infection of the mammalian host with the infective L3 stage. The glandular oesophagus of the L3 contains granules or inclusion bodies, which appear by electron microscopy to be similar in structure and density to the inner body. These contain a variety of products, which are released within 48 h of infection of the host. The inner body could be considered to be a counterpart of the glandular oesophagus in the infective stage for the vector. It is likely that the same group of cells is responsible for the synthesis of both structures. In the microfilariae, the pharyngeal thread and inner body are surrounded by the cells, which will go on to develop into the oesophagus and intestine [27]. After transmission and breakdown of the inner body, around the remains of the inner body there is a condensation of cytoplasm followed by numerous nuclear divisions, the cells resulting from these developing into parts of the intestine [33]. We have observed that between different species of filariae, composition of both the inner body and glandular oesophageal inclusions varies. In the case of chitinase, it is present in the *B. malayi* microfilariae but not in the L3 stage. The situation is reversed in *O. volvulus* where it is present in the L3 but not in the microfilariae. Our observation of chitinase in different inclusion body-like structures in the two different stages suggests that the inner body and the glandular oesophagus may originate from the same cell type, and that the inner body may be the precursor of the oesophageal gland. The divergence between species of filariae, possibly in response to their adopted lifestyles, could be a useful tool in elucidating the function of other inner body/glandular oesophageal components.
