# Supplementary figures and images for: Chitinase is stored and secreted from the inner body of microfilariae and has a role in exsheathment in the parasitic nematode Brugia malayi
Source: Mol Biochem Parasitol. 2008 Sep;161(1):55–62. doi: 10.1016/j.molbiopara.2008.06.007 (PMC2577134; doi:10.1016/j.molbiopara.2008.06.007)

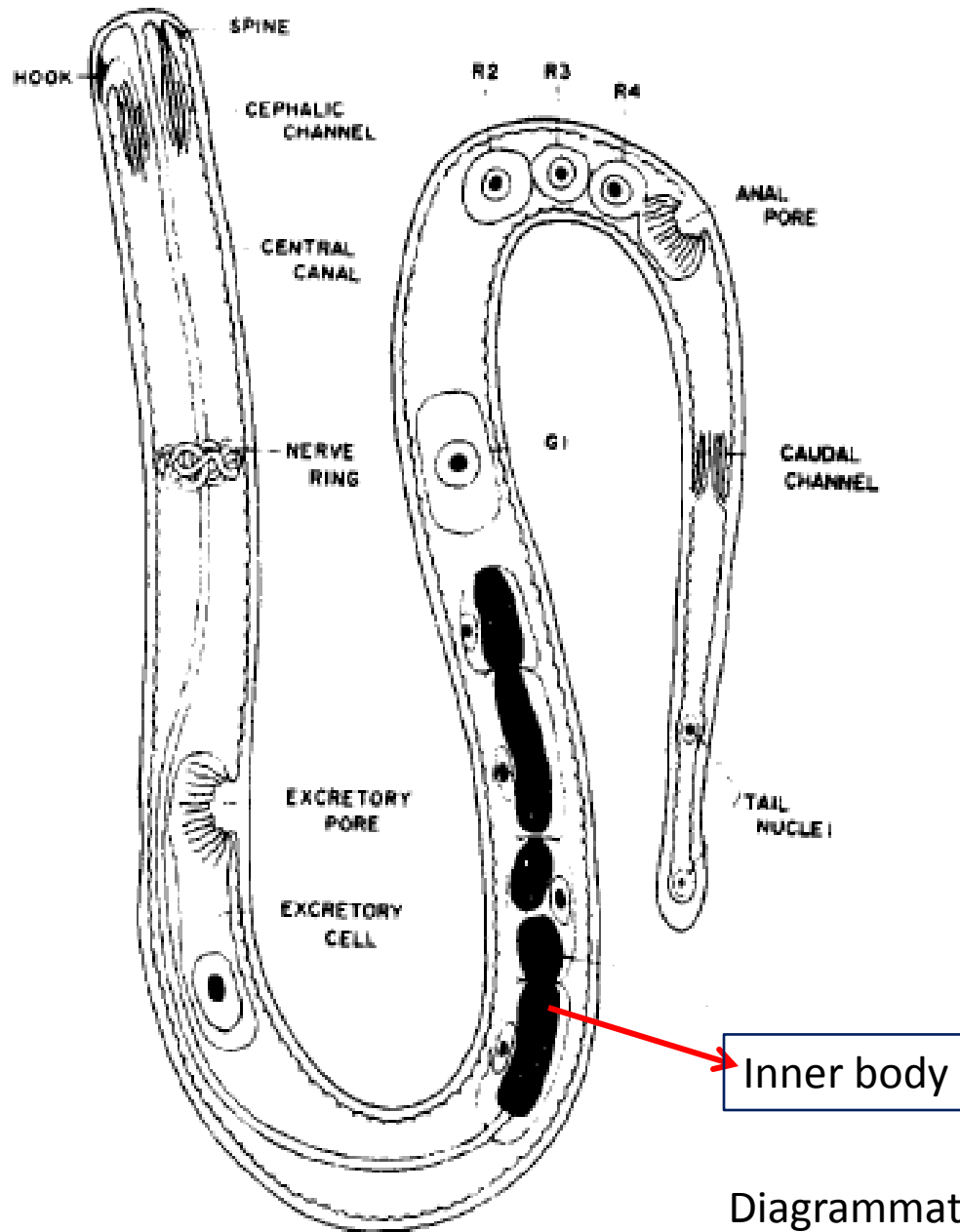

Diagrammatic drawing of  
*Microfilaria* from Tongu, 1974 ref.21

Supplement: Supplementary file 1 [file mmc1.pdf]
